# Use of Iodine-131 to Tellurium-132 Ratios for Assessing the Relationships between Human Inhaled Radioactivity and Environmental Monitoring after the Accident in Fukushima

**DOI:** 10.3390/ijerph15030483

**Published:** 2018-03-09

**Authors:** Koji Uchiyama, Masami Miyashita, Yoshinobu Tanishima, Shigenobu Maeda, Hitoshi Sato, Jun Yoshikawa, Shuji Watanabe, Masamichi Shibata, Shuji Ohhira, Gen Kobashi

**Affiliations:** 1 Laboratory of International Environmental Health, Center for International Cooperation, Dokkyo Medical University, 880 Kitakobayashi, Mibu-machi, Shimotsuga-gun 321-0293, Tochigi, Japan; s-ohhira@dokkyomed.ac.jp; 2 Laboratory of International Epidemiology, Center for International Cooperation, Dokkyo Medical University, 880 Kitakobayashi, Mibu-machi, Shimotsuga-gun 321-0293, Tochigi, Japan; 3 Technical Division, Anzai Medical Co., Ltd., 3-9-15 Nishishinagawa, Shinagawa, Tokyo 141-0033, Japan; 4 Department of Radiology, Fukui Prefectural Hospital, 2-8-1 Yotsui, Fukui-shi 910-8526, Fukui, Japan; m159miya@yahoo.co.jp (M.M.); y-tanishima-7v@pref.fukui.lg.jp (Y.T.); j-yoshikawa-ym@pref.fukui.lg.jp (J.Y.); s-watanabe-9e@pref.fukui.lg.jp (S.W.); mshiba37224@yahoo.co.jp (M.S.); 5 Emergency Medicine, Fukui Prefectural Hospital, 2-8-1 Yotsui, Fukui-shi 910-8526, Fukui, Japan; pxt01173@nifty.ne.jp; 6 Department of Radiological Sciences, Ibaraki Prefectural University of Health Sciences, 4669-2 Ami, Ami-machi, Inashiki-gun 300-0394, Ibaraki, Japan; satoh@ipu.ac.jp; 7 Department of Public Health, Dokkyo Medical University School of Medicine, 880 Kitakobayashi, Mibu-machi, Shimotsuga-gun 321-0293, Tochigi, Japan; genkoba@dokkyomed.ac.jp

**Keywords:** environmental monitoring, whole body counter measurement, iodine-131, tellurium-132, inhaled radioactivity, intake scenario, Fukushima accident, 24 h-thyroid uptake, physiological biokinetic model, thyroid autoregulation

## Abstract

Significant differences in findings were seen between the intake amounts of iodine-131 that were derived from direct measurements and the estimated intake from environmental monitoring data at the Fukushima accident. To clarify these discrepancies, we have investigated the iodine-131 and tellurium-132 body burdens of five human subjects, who after being exposed to a radioactive plume, underwent 21.5 h whole body counter measurements at Fukui Prefectural Hospital, so clear intake scenario and thyroid counter measurement data were available. To determine the iodine-131 and tellurium-132 body burdens, we introduced a new method of whole body counter calibration composed of a self-consistent approach with the time-dependent correction efficiency factors concept. The ratios of iodine-131 to tellurium-132, ranging from 0.96 ± 0.05 to 2.29 ± 0.38, were consistent with results of the environmental measurements. The 24 h iodine uptake values ranging from 12.1–16.0% were within euthyroid range in Japanese people. These results suggest, even if the relatively low thyroid iodine uptake in the Japanese population was taken into consideration, that there is no doubt about the consistency between direct measurements and environmental monitoring data. Adequate intake scenario is suggested to be principally important to estimate the inhaled radioactivity in areas in or around nuclear accidents.

## 1. Introduction

The assessment of internal exposure of emergency responders at nuclear accidents is important for evaluating the occupational doses of them as well as the effectiveness of countermeasures, such as pharmacologic thyroid blocking by oral potassium iodide. In addition, the results of the assessment with environment monitoring, at the same time and in the same place, are invaluable for evaluating radiation doses in the general population after nuclear accidents, and occasionally provides important and fundamental information about the intake of biokinetic radionuclides. Indeed, we previously reported that the thyroid iodine-131 (^131^I) activities derived from thyroid counter measurements were in good agreement with environment monitoring data, the results also implied a faster kinetics of iodine metabolism in younger adults as previously suggested by Hänscheid et al. [1,2].

Recently, however, a few groups have reported a difference between the intake amounts derived from direct measurements and the estimated intake from the air concentrations [3,4,5]. In their reports, estimated intake ratios of ^131^I to radiocesium (cesium-137 (^137^Cs) or cesium-134 (^134^Cs)) from in-vivo thyroid measurements were significantly smaller than the ratios derived from environmental measurements. They concluded that the estimated intake amounts of ^131^I from environmental monitoring data were overestimated because the transfer coefficient, 0.3, from blood to thyroid useby the International Commission on Radiation Protection (ICRP) in the biokinetic model was too high for the relatively low thyroid iodine uptake in the Japanese population. Meanwhile, Morita et al. reported that when the intake scenarios were taken into account, their internal radioactivity assessed by a whole body counter examination comparatively agreed with the predicted airborne radioactivity [6]. As regards to the intake scenario, Kurihara et al. and Kim et al. assumed an acute intake of 60% elemental iodine vapor and 40% particulate aerosols via inhalation on 15 March 2011 [3,4]; the assessments made by Hosoda et al., assumed a similar acute intake scenario using only inhalation of elemental iodine vapor [5]. Morita et al. also assumed acute inhalation, however, the date and duration of the particulate aerosols inhaled during the stay in Fukushima were specified by using each subject’s behavioral record [6]. All the groups used the same ICRP biokinetic model, hence the relationship between in-vivo measurements and environment monitoring data remained unclear at the Fukushima Daiichi Nuclear Power Plant accident.

To clarify these inconsistencies, we investigated the ^131^I and tellurium-132 (^132^Te) body burdens of the same subjects used in our previous report, whose intake scenario was clearly available [2]. Technically, the gamma-rays from ^132^I were strongly affected to determine ^134^Cs and ^137^Cs body burdens. Although the interference declines with time, it is an issue for early-stage measurements, these were shown by the measurements from the whole body counter that was equipped with NaI(Tl) detectors, immediately after the Fukushima accident [7]. Additionally, the gamma ray 637 keV from ^131^I might have an effect that may determine the ^134^Cs and ^137^Cs activities [8,9]. On the other hand, ^132^Te body burdens could be more clearly determined. The half-lives of ^131^I and ^132^Te are similar when compared to ^134^Cs or ^137^Cs, so that the estimated intakes from environment monitoring data could be less susceptible to intake scenarios if the ratios of ^131^I to ^132^Te were used. Therefore, the ratio of ^131^I to ^132^Te was better suited for this investigation than the ones of ^131^I to ^134^Cs or ^137^Cs. In consideration of the consistency of the thyroid ^131^I activities, previously reported by us, biokinetic calibration which will be described later in detail, was applied to the measurement data of the whole body counter to determine the ^131^I and ^132^Te body burdens. The consistency between the whole body counter measurements and several environment monitoring data are discussed using the ratios of ^131^I to ^132^Te and the 24-h thyroid uptakes.

## 2. Materials and Methods

### 2.1. Subjects and Ethical Considerations

The whole body counter measurements of five men of a six-member team that comprised the second disaster medical assistance team (DMAT) of Fukui Prefectural Hospital were used in the current study. The details of the subjects were described previously [2]. In short, four of five men ingested stable iodine pills, a total of 100 mg of potassium iodide, as prophylaxis for internal exposure at 02:30 on 14 March 2011. They were exposed to the radioactive plume at Tamura City in Fukushima Prefecture, from 13:00 to 15:00 on 15 March. The in-vivo measurements were performed in Fukui Prefectural Hospital at around 11:30 on 16 March. The current study was approved by the research ethics committees of Dokkyo Medical University (No. dmu25002) and Fukui Prefectural Hospital (No. 13-27). All the study participants provided written informed consent.

### 2.2. Whole Body Counter Measurements

**Figure 1:**
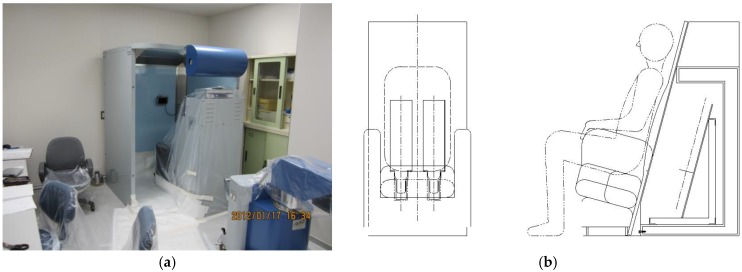
(**a**) Chair-type whole body counter at Fukui Prefectural Hospital. (**b**) Schematic drawings of the two NaI(Tl) detectors aligned alongside and horizontally in the chair.

**Figure 2:**
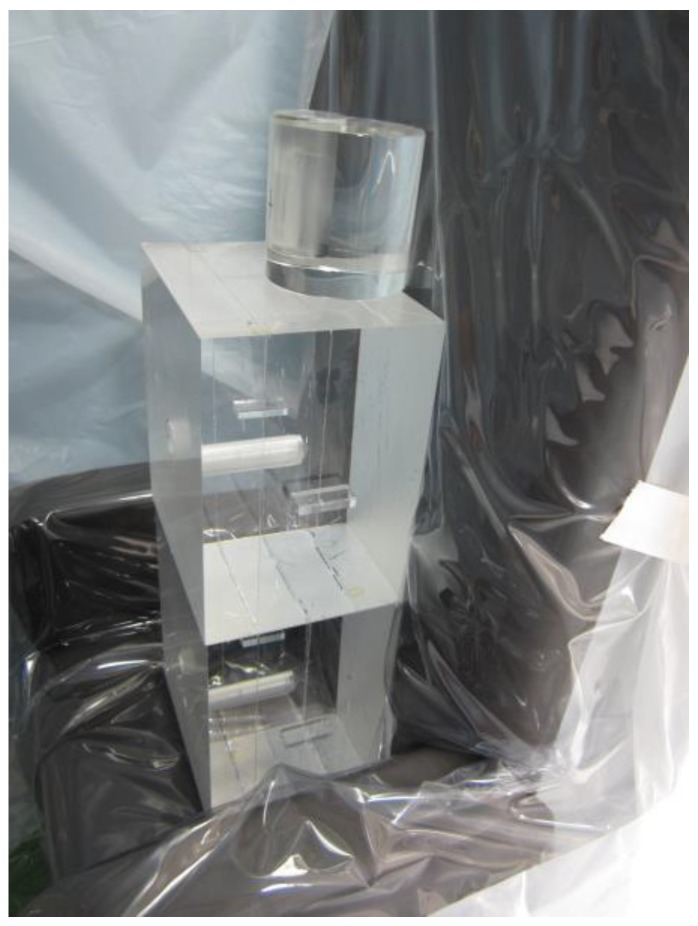
Thyroid phantom on the acrylic block phantoms.

**Figure 3:**
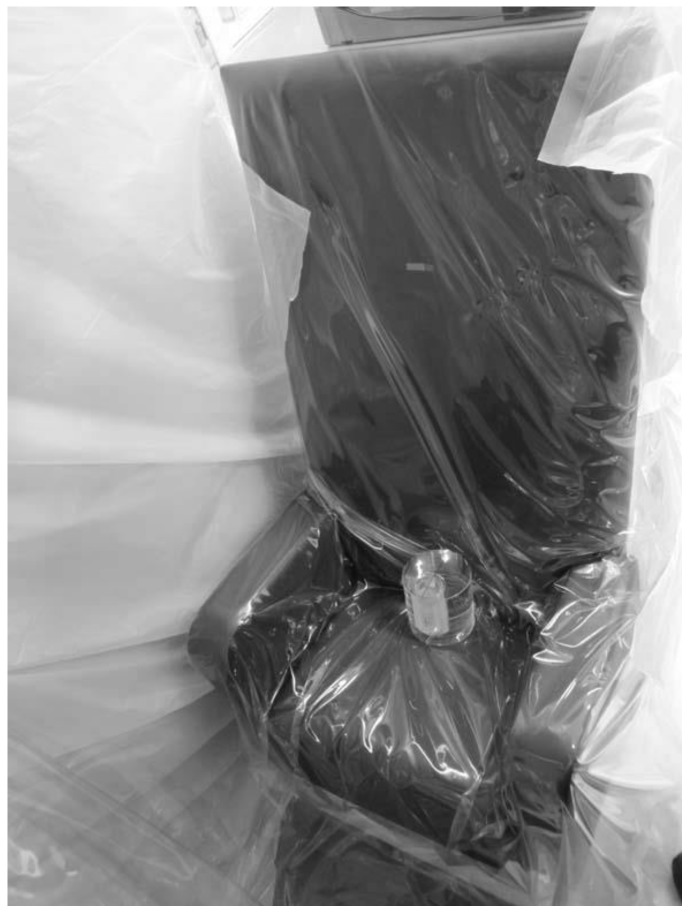
Substitute bladder phantom.

A chair-type whole body counter, a component of a whole body counting system (AZ-BC3, Anzai Medical Co., Ltd., Tokyo, Japan), equipped with two 7.6 × 12.7 × 40.6 cm NaI(Tl) detectors with a 30 mm lead allover shield was used (Figure 1). Calibration results of the bottle manikin absorption (BOMAB) phantoms [10,11] conducted by the National Institute of Radiological Sciences (NIRS) on October, 2007 (kindly provided by Dr. Takashi Nakano, NIRS) were used for homogeneous efficiency calibration by using the efficiency curve as follows [11]:

(1) $$\ln\left(\varepsilon \right)=a+b\;\ln\left(E\right)+c\;{\left(\ln\left(E\right)\right)}^{2}$$

where *ε* is the efficiency, *a*, *b* and *c* are constants, and *E* is the gamma ray energy (keV). Additionally, BOMAB phantoms made of acryl and polyvinyl chloride of a 10-year-old, an adult female and an adult male were manufactured and used for body size correction and background measurements. A mixture of barium-133 and ^137^Cs contained in a capsule (Mock iodine, MI501; Japan Radioisotope Association, Tokyo, Japan), with a thyroid uptake neck phantom (Neck Phantom, Thyroid Uptake; #043-365, Biodex Medical Systems, Inc., Shirley, NY, USA) [12,13] mounted on acrylic block phantoms were used for thyroid efficiency calibration (Figure 2). The mock iodine with the neck phantom was also used as a substitute phantom for the bladder (Figure 3). An europium-152 (^152^Eu) point source (EU401, Japan Radioisotope Association, Tokyo, Japan) with the acrylic block phantom was used to convert the efficiency of mock iodine into the efficiency of 228 keV gamma ray from ^132^Te, by means of the weighted least-squares fitting with Equation (1). The counting efficiency of 228 keV gamma ray, which was estimated from the peak efficiency curve by using 245, 344, and 779 keV, could be used in the efficiency conversion, because the energy difference between 228 and 245 keV was much smaller than the 12% energy resolution. Gnuplot version 4.6 [14] was used for this fitting.

A least-squares fitting with Gaussian functions and an exponential function, as the baseline function, was employed for analyses of the gamma ray spectra from the whole body counter measurements as previously described [2]. The data acquisition time for the 53-year-old man was 5 min; thereafter, only a 1 min acquisition time was used [2]. The 35-year-old man was processed twice because of a typing error of his body weight in the first measurement, therefore, 2-min acquisition data could be derived manually from the sum of the gamma ray spectra.

### 2.3. Biokinetic Calibration

The chair-type whole body counter we used was not designed to determine residual activity in a specific organ such as the thyroid, lung, bladder and so on. As biodistribution of iodine is inhomogeneous, to consider the previously determined residual activities in the thyroid for the subjects in this study, the biodistribution information that was estimated at the time of the measurements was incorporated into the calibration of the counting efficiencies of the whole body counter. This estimation was achieved by introducing a physiochemical compartmental treatment in the calibration. Namely, we performed a 3-pattern calibration to determine ^131^I body burdens as follows: first, the simplest, was the compartment calibration; hence the only BOMAB data was used for this homogeneous whole body calibration [7,15]. Second was two-compartment calibration, which presumed that the gamma rays came from the thyroid and homogeneous whole body (the rest of the compartments) independently. Third was biokinetic calibration, predominant three-compartment calibration, the thyroid, bladder (including urine), and homogenous whole body (the rest of the compartments), which was assigned by using the physiological biokinetic model proposed by Leggett [16]. We assumed the superposition principle, total ^131^I body burden *A_tot_* could be calculated by using the following formula:

(2) $$A_{tot}=\frac{1}{\frac{w_{thy}}{e_{thy}}\;+\;\frac{1\;-\;\left(w_{thy}\;+\;w_{wb}\right)}{e_{blad}}\;+\;\frac{w_{wb}}{e_{wb}}}C_{tot},$$

where *C_tot_* is the total counts derived from the whole body counter measurements, *e_thy_*, *e_blad_*, and *e_wb_* mean the efficiencies of the thyroid, bladder, and the rest (homogenous whole body), respectively. The residual activity ratios of the thyroid and the rest to the total body burden denote *w_thy_* and *w_wb_*, respectively. It should be noted that these residual activity ratios could only be determined from this three-compartment calibration, so that the biokinetic calibration automatically incorporated the effects of the iodine-rich Japanese diet. Regarding ^132^Te body burden, we used homogenous whole body calibration. However, for the sake of consistency, two-compartment calibration; the bladder and homogenous whole body, with the physiological biokinetic model proposed by Giussani [17], were also performed and used as the biokinetic calibration for ^132^Te with the biokinetic calibration for ^131^I.

To determine the residual activity ratios of each compartment to the total ^131^I body burden in the biokinetic calibration, self-consistent approach, together with the time-dependent correction efficiency factors concept [15] was applied. In this technique, an estimated thyroid residual activity of a subject was individually calculated repeatedly, using the physiological biokinetic model [16], until the result achieved was consistent with the thyroid counter measurement [2]. This self-consistency could be achieved by adjusting the transfer coefficient parameters in the three-compartment calibration, details of which are described later, also included are considerations and specific issues regarding Japanese people from a physiological point of view. Incidentally, in contrast, the ratios were automatically determined from the previous thyroid counter measurement results in the two-compartment calibration for ^131^I. We assumed that two transfer coefficients, *λ_1_* and *λ_5_*, which were predominantly affected by the influence of chronic iodine-rich diet, were variables. The coefficient *λ_1_* determines the fractional transfer rate of iodide from blood (Blood 1) to the thyroid (Thyroid 1), whereas the coefficient *λ_5_* indicates the fractional rate of “iodide leak” from the thyroid (Thyroid 2) to the blood (Blood 1) [16]. If necessary, the transfer coefficient from the urinary bladder contents to urine could also be used as a subsidiary variable. The other parameters were fixed with default values [16]. The duration of exposure to the radioactive plume was approximately 2 h [2]; therefore, we assume the absorption occurred quickly after inhalation at around 14:00 on 15 March 2011. The whole body counter measurements were conducted at around 11:30 on 16 March, so that the ^131^I biodistribution 21.5 h after inhalation was calculated and used in the biokinetic calibration. The respiratory tract was never considered, so that the estimated intake would be underestimated by the amount of exhaled particulate aerosols.

It is well established that the iodine uptake is mediated by the sodium-iodide symporter (NIS) of which expression and activity is predominantly regulated by I^−^ [18]. This thyroid autoregulation plays an important function, as it suggests that thyroid glands of Japanese subjects, on a diet rich in iodine, organify more iodine than they secreted as thyroid hormone and the excess is secreted as nonhormonal iodine [19]. In other words, rather than the downregulation of NIS-mediated iodine uptake, the iodide leak would be predominant when the biokinetic of persons, who are regularly receiving iodine sufficient diets, is considered. Using the Leggett model, the default parameters set provides the most reliable results [16]. Consequently, on the first step of the biokinetic calibration for ^131^I, adjusting *λ_5_* until the thyroid activity achieved a consistent with the thyroid measurement, 268 Bq for the 53-year-old man who was never administered stable iodine. The other parameters remained the default values. Specifically, the Leggett model was written as:

(3) $$\frac{dN_{i}\left(t\right)}{dt}=-\sum_{j}\lambda_{i\to j}N_{i}\left(t\right)+\sum_{j}\lambda_{j\to i}N_{j}\left(t\right),$$

where $N_{i}\left(t\right)$ is the iodine content in the compartment *i* at time *t*, $\lambda_{i\to j}$ denotes the transfer coefficient from compartment *i* to *j*. The calculation results with default parameters set provided *w_thy_* and *w_wb_* in the Equation (2). In parallel, a least-squares fitting as described above was performed to calculate *C_tot_* in the Equation (2). Then, the thyroid activity was derived from:

(4) $$A_{thy}=w_{thy}A_{tot}.$$

Until the $A_{thy}$ value is equal to the previously determined value, 268 Bq, recalculation of the Equation (3) with modified *λ_5_* was performed to achieve this value. Second, adjusting *λ*_1_ until the thyroid activity achieved a consistent with the thyroid measurement results of the other men who ingested stable iodine. In this step, we assumed that *λ*_5_ for the younger subjects were taken to be the same as that determined for the 53-year-old because of the thyroid autoregulation described above. These calculations were performed in order of age. If the calculations were indeterminate, they were aborted and restarted after modification of the transfer coefficient from the urinary bladder contents to urine of the younger subjects. If all calculations were completed, the ordering of *λ*_1_ and the consistency in the total body burdens would confirm each other. From our previous study, we assumed that *λ*_1_ was inversely related to age, except for the 53-year-old who had never taken the stable iodine pills [2]. Additionally, *λ*_1_ was assumed to be the same in the order of magnitude as the default value, 7.26 day^−1^. If the ordering was broken, the calculation was restarted from the first step after modifying the transfer coefficient from the urinary bladder contents to urine of the 53-year-old. If *λ*_1_ exceeded 9.3 day^−1^, this showed a relatively low dietary iodine model (Y/S = 2) [16], a renewed calculation was conducted after a modification of the transfer coefficient from the urinary bladder contents to urine of the 53-year-old man. The biokinetic calibrations for ^132^Te were conducted using the same transfer coefficients from the urinary bladder contents to urine in the biokinetic calibrations for ^131^I.

### 2.4. Nutritional Survey

To verify that the subjects were receiving sufficient iodine from their diets, we conducted nutritional interviews concerning their dispatch.

## 3. Results

### 3.1. Whole Body Counter Measurements

**Figure 4:**
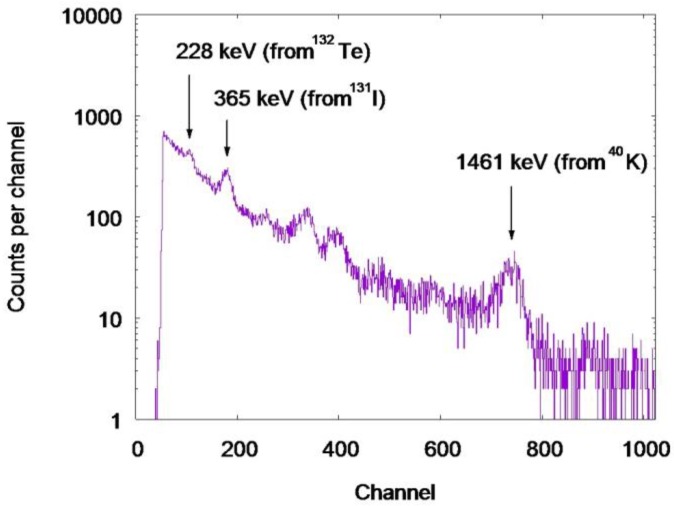
The gamma ray spectrum of the 53-old-year man, acquisition time = 5 min.

**Figure 5:**
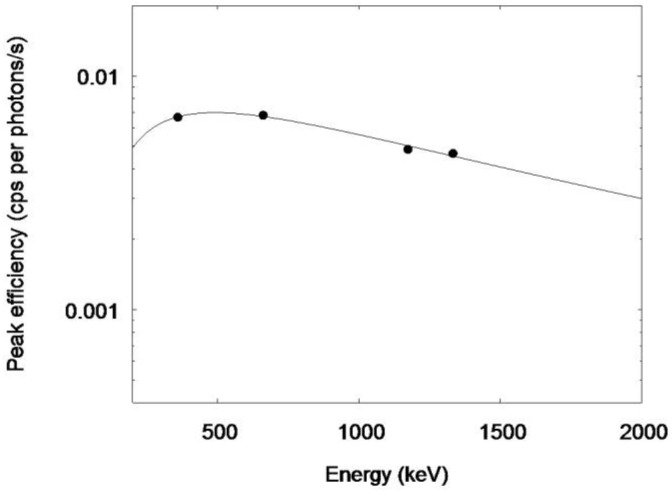
Efficiency curve derived from the NIRS BOMAB phantoms. Rigid circles indicate the measurement results. The solid line shows the least-squares fitting result.

**Figure 6:**
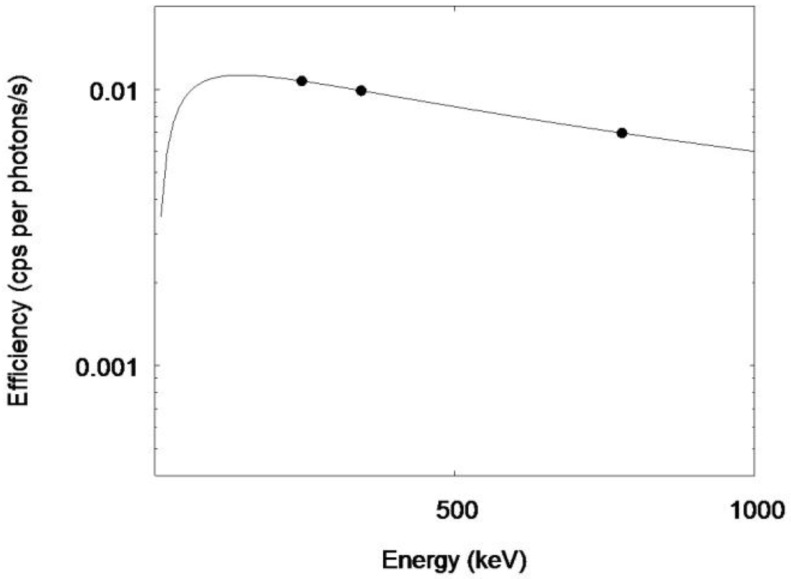
Efficiency curve derived from the ^152^Eu point source measurement. Rigid circles indicate calculated values from measurement. The solid line shows the weighted least-squares fitting result.

The gamma ray spectrum of the 53-year-old man is shown in Figure 4. The gamma ray peaks of 228 keV (^132^Te) and 365 keV (^131^I) are clearly seen. The multiple peaks in the middle of the spectrum consisted of the gamma rays from ^132^I, ^134^Cs, and ^137^Cs. The efficiency curve of the NIRS BOMAB phantoms is shown in Figure 5. The energy dependency of which shows a similar pattern to the other whole body counters in Fukushima [11]. Figure 6 shows the efficiency curve derived from ^152^Eu point source in the acrylic block phantom measurement.

The ^131^I residual activities in the thyroid to be preserved in estimations, which were determined in the previous study [2], are listed in Table 1. The ^131^I body burdens are summarized in Table 2 (homogenous and two-compartment calibration) and Table 3 (biokinetic calibration), respectively.

**Figure 7:**
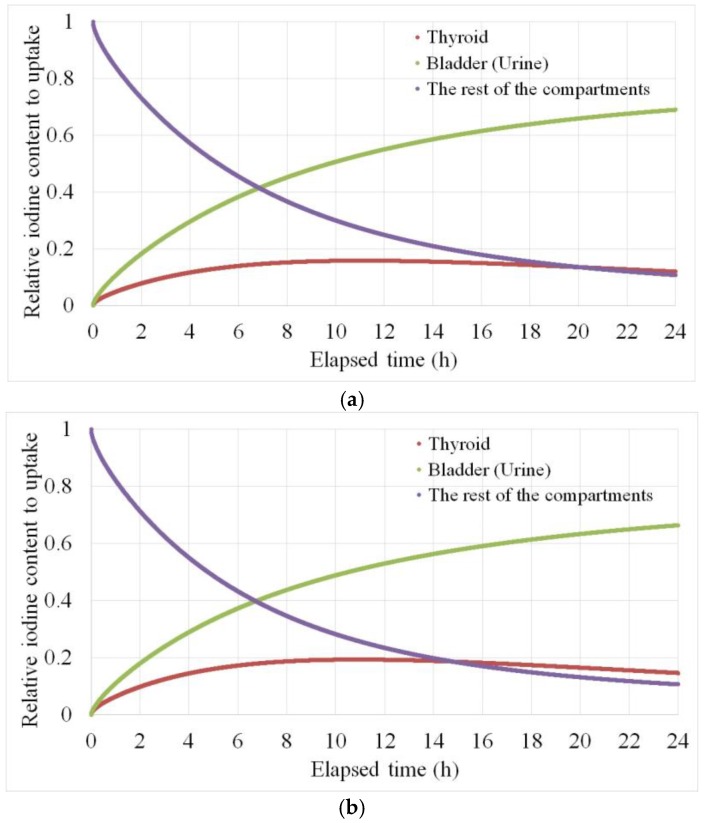
The estimated cumulative iodine changes of the three compartments for the (**a**) 53-year-old and (**b**) 27-year-old men.

We consider that the statistical errors from the gamma ray peaks are the only uncertainties in this study. The 24-h thyroid uptake could not be defined in the case of the homogenous calibration, avoiding contradiction, whereas the maximum values could be estimated in the two-compartment calibration. In contrast, the 24-h thyroid uptake values could be directly derived in the biokinetic calibration, and is shown in the table with the adopted variables. The 24-h thyroid uptakes of the subjects were within 0.12–0.16, which were in good agreement with the normal Japanese range, 0.12–0.25 [4,20]. The estimated cumulative iodine changes of the compartments for the 53-year-old and 27-year-old men are shown in Figure 7. In the calculation of the biokinetic models, the differential Equation (3) were transformed into difference equations as shown in the following expression:

(5) $$N_{i}\left(t+\Delta t\right)=N_{i}\left(t\right)+\left(-\sum_{j}\lambda_{i\to j}N_{i}\left(t\right)+\sum_{j}\lambda_{j\to i}N_{j}\left(t\right)\right)\Delta t.$$

After some tests, such as reproducing the published results [16], 0.01 h was used as Δt value. The ^132^Te body burdens were shown in Table 4.

The ratios of ^131^I to ^132^Te were calculated from the estimated total uptakes of ^131^I and ^132^Te derived from the biokinetic calibrations for ^131^I and both for ^132^Te, and then the decay corrected values to 17:00 on 15 March 2011 are summarized in Table 5. The arithmetic and weighted averages of the ratio of ^131^I to ^132^Te derived from the subjects were 1.63 ± 0.55 and 1.02 ± 0.05 from the biokinetic calibration (^131^I and ^132^Te), and 2.02 ± 1.32 and 1.00 ± 0.05 from the biokinetic (^131^I) and homogenous (^132^Te) calibrations.

### 3.2. Nutritional Survey

The contents of the meals during the dispatch, including the stable iodine pills, are summarized in Table 6. Wakame (seaweed) is a well-known iodine-rich food. Kombu (seaweed) extract is typically included in miso soup and tempura udon as well as in Akai kitsune udon (instant noodles), these are also good sources of iodine. Overall, all the subjects had taken sufficient iodine from their diets, including the 53-year-old man who never took the stable iodine pills.

## 4. Discussion

In the present study, we have determined the ^131^I and ^132^Te body burdens of five men of a six-member team that comprised the second DMAT of Fukui Prefectural Hospital. The biokinetic calibration was introduced to consider the thyroid autoregulation that plays an important role on the subjects who are on a rich iodine diet. Using the biokinetic calibration results, the arithmetic and weighted averages of the ratio of ^131^I to ^132^Te derived from the subjects were 1.63 ± 0.55 and 1.02 ± 0.05 from the biokinetic calibration ^131^I and ^132^Te, and 2.02 ± 1.32 and 1.00 ± 0.05 from the biokinetic (^131^I) and homogenous (^132^Te) calibrations.

In the previous study, the soil measurement was performed at Tokiwa Junior High School, which is approximately 4 km to the east of the Tamura City Sports Park, the decay corrected ratio of ^131^I to ^132^Te was 1.92 ± 0.03 [21]. On the other hand, the air dose rate measurement that was conducted at 15:51 on 15 March 2011 in the Abukumakogen Service Area (SA), which is approximately 6 km to the south of the Tamura City Sports Park, the decay corrected ratio of ^131^I to ^132^Te was 0.78 [22]. In the present study, the arithmetic and weighted averages of the ratio of ^131^I to ^132^Te derived from the subjects were 1.63 ± 0.55 and 1.02 ± 0.05 from the biokinetic calibration ^131^I and ^132^Te, and 2.02 ± 1.32 and 1.00 ± 0.05 from the biokinetic (^131^I) and homogenous (^132^Te) calibrations. Here, we assume that the physicochemical form of ^131^I was 60% elemental iodine vapor and 40% particulate aerosols (absorption type F) [4] and the physicochemical form of ^132^Te was 100% particulate aerosols (absorption type F). Information regarding the rate of absorption in the lungs for ^132^Te was not available, so that the deposited ^132^Te in the lungs could have remained there [23]. Therefore, in this case, we should adopt the ratios from the biokinetically calibrated ^131^I to the homogenous calibrated ^132^Te, because the homogenous calibration results included the total residual activities in the lungs independent of the physicochemical forms. We never considered the respiratory tract, the factors 0.8 (=0.4 from aerosol mixing rate times 0.5 from exhaled correction plus 0.6 from vapor) for ^131^I and 0.5 for ^132^Te were smaller than the ratio [4], this can be compared to the air concentration data from the environmental monitoring. As the result, arithmetic and weighted averages of the ratio of ^131^I to ^132^Te were 1.26 ± 0.83 and 0.63 ± 0.03, where 1.26 = 2.02/(0.8/0.5) and 0.63 = 1.00/(0.8/0.5), respectively. Therefore, the result was in good agreement with the result of the air dose rate measurement at Abukumakogen, SA. Although, our result was slightly lower, it was not inconsistent with the soil measurement collected at Tokiwa Junior High School. Accordingly, this result supports that environmental measurements could be applied for estimating the intake amounts of the human subjects as in the past, even if the diet of the subjects customarily includes moderate to large quantities of foods rich in iodine.

The estimated total uptake of ^131^I was not significantly dependent on the models, within a range of 1000 to 7000 Bq; however, each value fluctuated within 0.4- to 2.1-fold range. Morita et al. reported their whole body counter was calibrated by using the BOMAB phantoms, so that their results seemed to be homogenously calibrated [6,7]. They mentioned the results of the difference of the ^131^I/^137^Cs ratios between their human measurements and the Worldwide Version of System for Prediction of Emergency Dose Information (WSPEEDI-II) simulations, which predicted the spatiotemporal distribution of activity concentrations in ground-level air from estimated release rates of radionuclides from the reactors. Whereas the accrual internal radioactivity assessed by the whole body counter examination comparatively agreed with the predicted airborne radioactivity by the WSPEEDI-II simulation. Our results suggest that the difference of the ^131^I/^137^Cs ratios may be caused by the calibration model. We should also mention that, as described in the introduction section, earlier measurements by Morita et al. might be influenced by the overlap of gamma ray peaks from ^132^I and ^134^Cs, and ^132^I and ^137^Cs. This may have contributed to the discrepancy.

The inconsistency in the direct comparison between the in-vivo measurements and the soil sampling data was pointed out by Hosoda et al. [5]. In the present study, the deposition density 5.33 × 10^2^ kBq/m^2^ derived from the soil measurements at Tokiwa Junior High School by Endo et al. [21] could be a comparable result of soil measurements. Under the assumption that the deposition was dry, i.e., the deposition rate was 10^−3^–10^−2^ m/s, the estimated time-integrated concentration in air could be evaluated to be in the range of 5.33 × 10^4^ to 5.33 × 10^5^ kBq s/m^3^. The other comparable data are from the United States National Oceanic and Atmospheric Administration (NOAA) simulation results published in the UNSCEAR 2013 report, which estimated dispersion and deposition from reverse modelling of environmental measurements of concentrations in the air, deposition densities, dust samples, and air dose rates [24]. The NOAA estimated values of the deposition density and the time-integrated concentration in the air at Denso Higashinihon, which is approximately 10 km west of the Tokiwa Junior High School, were 3.6 × 10^2^ kBq/m^2^ and 9.4 × 10^3^ kBq s/m^3^, respectively. The results of the deposition density corroborate in both cases; however, the estimated time-integrated concentrations in the air were significantly different. When compared to the present results, the NOAA simulation results are likely to be a better estimation because the inhalation intake was calculated 3.9 kBq under the assumption that the duration of the deposition was the same as the time of exposure to the radioactive plume (2 h) and the ventilation rate was 1.5 m^3^/h [2,25]. This assumption is consistent with Morita et al. that the accurate estimation of internal doses in the first week after the radiological accident was critical [6]. The soil was sampled not less than 12 h after the subjects in this study withdrew from the area. The weather data of Japan Meteorological Agency showed that it rained at midnight [26,27] after they left. Consequently, the additional wet deposition should be considered when using the soil measurement data. Meanwhile, the air concentration data estimated by an atmospheric dispersion model, such as the one used by NOAA, are comparatively robust to the intake scenario of the human subjects, so that the adjusted integration period, to match the intake scenario of the subjects, may provide a better estimation value, at the same time, the deposition density were in good agreement with both results.

Results of in-vivo measurements ^131^I, and environment monitoring, coinciding in time and place, were quite limited. To our knowledge, excluding our previous study, only one report from Kurihara et al. is available [3]. They estimated that the intakes of three workers were 3990–6190 Bq which were derived from the thyroid ^131^I residual activities, 183–285 Bq, under the assumption of acute inhalation on the morning of 15 March. The air dose rate at the time of exposure to the radioactive plume at their location was increasing from 0.25 to 4.8 μGy/h during 3 h and the atmospheric ^131^I concentration from the air sampling results at that time was 1.6 kBq/m^3^ [3,28]. The atmospheric concentration *C_A_* can be calculated by using the following formula [29]:

(6) $$C_{A}=\frac{I_{0}}{B\tau },$$

where *I*_0_ represents the intake, *B* is the individual’s breathing rate, and τ is the exposure duration. As the result, we should note that the estimated atmospheric ^131^I concentrations of 1.4–2.2 kBq/m^3^ which were obtained from the thyroid measurements, with the daily-averaged ventilation volume for adult males 0.925 m^3^/h [3], agreed very well with the air sampling measurement. These results imply that there is little doubt about the consistency between the in-vivo measurements and the air sampling results. On the other hand, in the present study, the estimated total uptake of ^131^I is within the range of 2000–7000 Bq and the air dose rate at the exposed to the radioactive plume was around 2.2 μGy/h [2]. The relationship between ^131^I intake and air dose rate was in good agreement with both the Kurihara et al. results and the present study. Consequently, these results may indicate that the atmospheric ^131^I concentration from either the results of actual measurements, such as air sampling or of the simulation based on the environment monitoring data, could provide optimal estimations of the inhaled ^131^I body burdens. Additionally, the air dose rate is likely to be a more useful indicator of the ^131^I inhaled amounts at the Fukushima accident.

The conventional estimate of the protective effect of pharmacologic thyroid blockage by using stable iodine pills is 70% [2], however this could be estimated to be about 10% if the reduction of the coefficient *λ*_1_ occurs by the downregulation of NIS-mediated iodine uptake. This relatively low protective effect might be the result of the faster kinetics caused by the thyroid autoregulation [18,19]. Further investigation in the internal radiation dosimetry may be warranted to assess how many doses would be influenced by this phenomenon, a premature ingestion of the stable iodine may cause a reduction of its protective effect on the persons receiving regular iodine sufficient diets. This result suggests that the timely decision based on the change of the air dose rate may be essential and be more effective in the prophylaxis of the stable iodine administration, as conducted in Miharu town, which is a neighboring town of Tamura City [30].

We acknowledge that there are several limitations in this study. First, the biokinetic calibration was performed phenomenologically. Indeed, several possible parameter sets exist when only self-consistency on each subject is required. Because there was no information about the relationships between age and NIS-mediated iodine uptake (*λ*_1_) or iodide leak (*λ*_5_), this could suggest the possibility that several separate parameter sets could apply to each subject. However, the parameter set we adopted was fully considered as systematic consistency, such as age ordering of *λ*_1_, within the normal Japanese range of 24-h thyroid uptake, and agreement with each other in estimated absorption. In addition, minimal parameter changes from the default values were conducted. The potential importance of the model is to show that accounting of the iodide leak is the key in obtaining a rational explanation for every relationship between the in-vivo thyroid measurements and the environment monitoring results even if the thyroid uptake in Japanese populations is relatively low, as discussed above. Second, the influence of the stable iodine prophylaxis and nutritional iodine intake does not reflect the biokinetic calibration. However, generalized parameter value of *λ*_5_ is yet to be known. Unusual modifications were needed for the transfer coefficient from the urinary bladder contents to urine, causing large differences in the total body burdens in Table 2 and Table 3. Meanwhile, it is well known that urinary iodine excretion exhibits large variations [31], and recently an intravesical urine vanishment phenomenon has been reported [32]. The estimated urinary excreted ^131^I was about 1000–5000 Bq, which was in the range of 0.2–1.1 pg in 21.5 h. This was negligibly small compared to the amount of the stable iodine excretion at the same time, which was in the range of 10 μg to 10 mg per 24 h in general [31]. These arguments may not be sufficient, and further developments, such as the extension of the range of the Leggett model application to children, were recently reported [33]. Therefore, further research in this area, especially to determine *λ*_5_ value for the subjects who are regularly receiving iodine sufficient diets, is warranted. Third, the effect of shelter-in-place had never been considered. As described previously, the team advised the residents to take refuge, as soon as possible, immediately after they were aware of the sudden increase of the air dose rate [2]. It is also possible that the amount of exposure to the subjects depended on where the team members were: in the gymnasium hall or outside [2]. This might be caused in part to the dispersion of the total body burdens. Finally, the respiratory tract was never considered, so that the estimated intakes reported are realistic absorbed amounts. However, the physicochemical form of the inhaled iodine is not clear, and the estimated intakes were in good agreement with the previous study as shown above. Further investigations are needed.

## 5. Conclusions

There are no doubts about the consistency between the in-vivo measurements and the environment monitoring, especially the ^131^I air concentration data, even if the relatively low thyroid iodine uptake in Japanese populations is considered. The thyroid autoregulation may play a key role of the thyroid metabolism, so the reported discrepancy between the in-vivo measurements and the environment monitoring data previously may be caused by assumptions of inadequate intake scenarios.

## Figures and Tables

**Table 1:** The ^131^I residual activities in the thyroid to be preserved in estimations.

Age (Year)	^131^I Residual Activity in the Thyroid (Bq)
53	268
49	249
35	676
34	569
27	1082

**Table 2:** Measurement of total body burden values; estimated total uptake and 24-h thyroid uptake of ^131^I, estimated from homogenous and two compartments calibrations. The total body burden was at 11:30 on 16 March 2011.

Age (Year)	Calibration Model	Total Body Burden (Bq)	Estimated Total Uptake (Bq)	24-h Thyroid Uptake (%)
53	Homogenous	3927 ± 135	4243 ± 146	-
	Thyroid and others	3716 ± 128	4015 ± 138	<7.2 ± 1.1
49	Homogenous	2321 ± 165	2508 ± 178	-
	Thyroid and others	2320 ± 165	2507 ± 178	<10.7 ± 3.8
35	Homogenous	3918 ± 127	4233 ± 137	-
	Thyroid and others	4050 ± 131	4376 ± 142	<16.7 ± 2.7
34	Homogenous	1284 ± 182	1387 ± 197	-
	Thyroid and others	1140 ± 162	1232 ± 175	<49.9 ± 11.0
27	Homogenous	2623 ± 195	2834 ± 211	-
	Thyroid and others	2454 ± 182	2652 ± 197	<44.1 ± 5.8

**Table 3:** Measurement of total body burden values; estimated total uptake, and 24-h thyroid uptake of ^131^I, estimated from biokinetic calibrations with adopted variable parameters. The total body burden was at 11:30 on 16 March 2011.

Age (Year)	Blood 1 to Thyroid 1 (*λ*_1_) (day^−1^)	Thyroid 2 to Blood 1 (*λ*_5_) (day^−1^)	Urinary Bladder Contents to Urine (day^−1^)	Total Body Burden (Bq)	Estimated Total Uptake (Bq)	24-h Thyroid Uptake (%)
53	7.26	1.50	0	1912 ± 254	2065 ± 274	13.1 ± 1.7
49	6.65	1.50	0	1913 ± 159	2066 ± 172	12.1 ± 1.0
35	9.20	1.50	0	3955 ± 131	4272 ± 141	15.9 ± 0.5
34	9.25	1.50	12	1089 ± 155	3589 ± 511	16.0 ± 2.3
27	9.25	1.50	6.2	2277 ± 175	6829 ± 525	16.0 ± 1.2

**Table 4:** Measurement of total body burden values, and estimated total uptake of ^132^Te, derived from the homogenous and the biokinetic calibrations. The total body burden was at 11:30 on 16 March 2011.

Age (Year)	Calibration Model	Total Body Burden (Bq)	Estimated Total Uptake (Bq)
53	Homogenous	1856 ± 166	2253 ± 202
	Biokinetic	1249 ± 112	1516 ± 136
49	Homogenous	1331 ± 202	1616 ± 245
	Biokinetic	1223 ± 185	1485 ± 225
35	Homogenous	3697 ± 155	4488 ± 188
	Biokinetic	3744 ± 157	4545 ± 191
34	Homogenous	886 ± 233	1075 ± 283
	Biokinetic	868 ± 229	1743 ± 460
27	Homogenous	1627 ± 237	1975 ± 288
	Biokinetic	1576 ± 230	3025 ± 441

**Table 5:** The decay corrected ratios of ^131^I to ^132^Te to 17:00 on 15 March 2011.

Age (Year)	Ratio of ^131^I to ^132^Te	
	Biokinetic Calibrations (^131^I and ^132^Te)	Biokinetic (^131^I) and Homogenous (^132^Te) Calibrations
53	1.38 ± 0.22	0.93 ± 0.15
49	1.41 ± 0.24	1.30 ± 0.22
35	0.96 ± 0.05	0.97 ± 0.05
34	2.09 ± 0.63	3.39 ± 1.01
27	2.29 ± 0.38	3.51 ± 0.58

**Table 6:** The content of meals during dispatch, including the ingestion time of the stable iodine pills.

Time and Date	Menu
Between 15:30 on 13 March and 2:00 on 14 March	Bread, rice ball, and “CalorieMate”
2:30 on 14 March	Stable iodine pills, 100 mg (4 young members)
7:15 on 14 March	Rice, miso soup (with Welsh onion), scrambled egg (with ketchup), sliced pork and salad (bean sprouts and lettuce)
Around 13:00 on 14 March	Bread and rice ball
21:15 on 14 March	Curry and rice, and instant noodles
7:00 on 15 March	Rice, miso soup (with sea weed and tofu), Japanese omelette, and Japanese pickles (napa cabbage)
12:30 on 15 March	Cup noodle (Akai kitsune udon), white bread (with mayonnaise), rice ball and Kashi Pan (bread with sweet filling)
19:20 on 15 March	Tempura udon, and rice ball
